# Intermittent hypoxia: linkage between OSAS and epilepsy

**DOI:** 10.3389/fphar.2023.1230313

**Published:** 2023-11-23

**Authors:** Yue Ma, Qiong Wu

**Affiliations:** ^1^ Department of Pulmonary and Critical Care Medicine, Shengjing Hospital of China Medical University, Shenyang, China; ^2^ Department of Neurology, Shengjing Hospital of China Medical University, Shenyang, Liaoning, China

**Keywords:** OSAS, epilepsy, intermittent hypoxia, inflammation, ER stress

## Abstract

Obstructive sleep apnea syndrome (OSAS) refers to the current apnea during sleep caused by upper airway collapse. Meanwhile, epilepsy is a common neurological disorder with a tendency for spontaneous and persistent seizures. Accumulating evidence indicates that OSAS was not independent of epilepsy. Patients with OSAS were observed to be susceptible to epilepsy, while OSAS could decrease the seizure threshold in epilepsy. However, the mechanisms underlying the association of OSAS with epilepsy have not been fully understood. In this study, we propose that intermittent hypoxia, common among OSAS patients due to upper airway collapse, is the linkage between OSAS and epilepsy. Intermittent hypoxia induces elevated levels of oxidative stress and inflammation, potentially causing excessive inflammatory and endoplasmic reticulum stress in brain tissue, which might ultimately lead to the development of epilepsy. Therapeutic approaches targeting inflammation and oxidative stress may provide novel insights into the treatment of OSAS and epilepsy.

## 1 Introduction

Obstructive sleep apnea syndrome (OSAS) is a common sleep-related breathing disorder which is characterized by recurrent upper airway collapse during sleep causing apnea, followed by arousal that re-establishes the airway (Lv et al., 2023). The severity of OSAS is differentiated clinically by the number of apnea-hypopnea events per hour of sleep and the apnea-hypopnea index (AHI) (Sateia, 2014; Kapur et al., 2017). Apnea is characterized by a 90% reduction in airflow lasting for at least 10 s, whereas Hypopnea is defined as a decrease in airflow of at least 50%, coupled with a 3% drop in blood oxygen saturation, lasting for a minimum of 10 s (Louter et al., 2012; Virk and Kotecha, 2016). OSAS arises from a mix of structural and neuromotor factors causing upper airway narrowing and collapse during sleep, disrupting sleep patterns (Schwab, 2003; Ruttanaumpawan et al., 2009; Marcus et al., 2017). Among OSAS patients, inadequate ventilation caused by apnea leads to insufficient air exchange, resulting in hypercapnia (PaCO2 > 45 mmHg), which prompt the patients to awaken, and facilitate the restoration of airway patency (Kaur et al., 2020). However, the resulting sleep fragmentation could contribute to various cognitive disorders such as concentration issues, excessive daytime sleepiness (EDS), and fatigue (Ferguson et al., 2017). In addition, it is common for OSAS patients to experience symptoms such as morning bloating, headaches, emotional numbness, depression, irritability, mood fluctuations, memory problems, and erectile dysfunction during the daytime (Azagra-Calero et al., 2012; Lévy et al., 2015). OSAS is a highly prevalent disorder around the world with considerable morbidity and mortality (Batool-Anwar et al., 2014). In the United States, about 12% of men and 7% of women aged 30–70 suffer from moderate to severe OSAS (Peppard et al., 2013). It was also observed in Brazil that 24.8% of men had OSAS (Tufik et al., 2010). In addition, OSAS is also common in China and Spanish, with prevalence of 9.5% and 14.2% correspondingly (Durán et al., 2001; Li et al., 2005). Recent studies also estimated that about 936 million people between 30 and 60 years old are affected by OSAS (Benjafield et al., 2019). Obstructive events predominantly occur during the rapid eye movement (REM) sleep stage (Haba-Rubio et al., 2005). During REM sleep, the hypoglossal nerve is inhibited through cholinergic neurons, resulting in the suppression of genioglossus muscle tone and an elevated risk of upper airway collapse (Mokhlesi et al., 2014). Collapse of upper airway further exacerbates the severity of OSAS and lead to blood hypoxemia, hypercapnia, sleep fragmentation, enhanced respiratory effort, which subsequently result in increased risks of intermittent hypoxia during sleep (Rosenzweig et al., 2014; Xing et al., 2014; Xanthopoulos et al., 2015). Long-term intermittent hypoxia could further result in a series of changes in the body, including oxidative stress, obvious inflammation, apoptosis, and neural activation (Huang et al., 2018; Patke et al., 2020; Mishra et al., 2021). Meanwhile, increased sympathetic activity during REM sleep also results in multiple systems such as cardiovascular system and nervous systems (Mokhlesi et al., 2014; Grigg-Damberger and Foldvary-Schaefer, 2021; Lehner et al., 2022). Therefore, OSAS patients might also have vario`us other medical conditions concomitantly.

Epilepsy is a common neurological disorder with a tendency of spontaneous and persistent seizures characterized by recurrent epilepsy, which will bring adverse effects on patients’ cognitive, psychological, and social life (Engel, 1995; Fisher et al., 2014). The defining feature of epilepsy is the presence of spontaneous recurrent seizures, which at a cellular level manifest as aberrant discharges of neuronal clusters leading to hyperexcitability and hypersynchrony (Ibhazehiebo et al., 2018). More than 70 million people around the world suffer from epilepsy (Thijs et al., 2019). The prevalence of epilepsy varies globally, with rates of approximately 0.4% in China, 0.39% in India, 0.3% in Europe, and 0.93% in Africa (Forsgren et al., 2005; Mac et al., 2007; Ba-Diop et al., 2014). In addition, it was also observed in France in 2020 that the incidence of epilepsy in France is about 1.02% (Coste et al., 2023). A seizure is a temporary sign or symptom triggered by abnormal, excessive, or synchronized neuronal activity in the brain. It can potentially cause permanent neuronal damage, leading to lifelong neurological problems. (Fisher et al., 2005; Parfenova et al., 2012). According to the latest classification methods of epilepsy, epilepsy is mainly divided into the following types: generalized epilepsy syndromes, with polygenic etiologies; self-limited focal epilepsy syndromes with presumed complex inheritance; self-limited focal epilepsy syndromes with presumed complex inheritance; a combined generalized and focal epilepsy syndrome with polygenic etiology, epilepsy syndromes with developmental encephalopathy (Riney et al., 2022).

More and more studies have shown that OSAS and epilepsy often coexist (Ebben et al., 2008; Parhizgar et al., 2011; Shaki et al., 2011). As is mentioned above, recurrent apnea during sleep and sleep fragmentation in OSAS patients could result in intermittent hypoxia in various tissues especially the brain. Indeed, OSAS patients frequently undergo abnormal physiological consequences in brain tissues due to prolonged oxygen deficiency (Burnsed et al., 2019; Li et al., 2021). As the brain is more sensitive to hypoxia injuries, intermittent hypoxia can cause more serious brain damage compared with other pathological changes (Rodgers et al., 2016). It was further demonstrated by Wang *et al.* that activities of GABAA receptor is modified by hypoxia, which further exacerbates brain injuries and increased the risk of epilepsy (Wang and Greenfield, 2009). When hypoxia is corrected through continuous positive airway pressure ventilation, epileptic seizures are controlled in patients with OSAS, further confirming the connection between intermittent hypoxia and epilepsy (van Golde et al., 2011). In addition, intermittent hypoxia, especially during REM stage, could also lead to high levels of inflammation and oxidative stress (Drum et al., 2016; Cronstein and Sitkovsky, 2017). Inflammation and oxidative stress in brain tissues could contribute to neuronal cell damage, which subsequently increased the risks of epileptic seizures. Moreover, it was also demonstrated by Lehner J *et al.* that sleep deprivation, common among OSAS patients, exhibited as a strong trigger for seizures (Lehner et al., 2022). Thus, it is possible that intermittent hypoxia caused by OSAS may play a significant role during the pathogenesis of epilepsy. In this study, we illustrate the mechanisms of intermittent hypoxia caused by OSAS, and further elucidate that inflammation and oxidative stress induced by intermittent hypoxia act as a bridge linking OSAS and epilepsy (Kong et al., 2012; Na et al., 2020).

## 2 Methods and materials

### 2.1 Objectives

The aim of the present study was to investigate the relationship between epilepsy and further identify underlying mechanisms.

### 2.2 Hypotheses

Intermittent hypoxia caused by OSAS, especially during REM sleep, might play crucial role in the pathogenesis of epilepsy.

### 2.3 Methods, key words, and search strategy

PubMed, PubMed Central (PMC), Embase, and Web of Science databases were searched for this study, using the following keyword: “obstructive sleep apnea syndrome or OSAS,” “REM sleep,” “epilepsy,” “intermittent hypoxia,” “oxidative stress or ROS,” “inflammation,” “TNF-α or IL-1β or IL-6,” “endoplasmic reticulum stress or ER stress” and “treatment.” The search was limited to English-language publications. Searches of published papers were conducted up to 18 October 2023. In addition, references from relevant articles were also examined to identify additional eligible studies.

## 3 OSAS and epilepsy

Common clinical manifestations of OSAS include symptoms of upper airway obstruction, insomnia, and sleep-related apnea. The occurrence of sleep apnea in patients with OSAS is due to the collapse of the upper airway during sleep, and airway stenosis is an anatomical factor for sleep apnea (Kim et al., 2012; Schloss et al., 2020). However, OSAS is not only a respiratory disease, but also a long-term disease process that can affect the circulatory system, nervous system and even psychology of patients (Milagro et al., 2019). It was noted that OSAS and epilepsy often coexist and influence each other. Many studies have shown that the incidence of OSAS is higher in patients with epilepsy (Maurousset et al., 2017). The prevalence of OSAS in patients with epilepsy is higher than that in the general population (van Golde et al., 2011; Shaheen et al., 2012). A cohort study published in 2018 revealed that patients with refractory epilepsy and OSAS experienced more frequent focal seizures and a longer duration of epilepsy, indicating a stronger association between OSAS and focal seizures (McCarter et al., 2018). Furthermore, it was also noted that the treatment for OSAS can alleviate symptoms of epilepsy. In a case report in 1994, continuous positive nasal pressure (CPAP) was given to 7 patients with both refractory epilepsy and sleep apnea syndrome, showing that 4/5 of the patients significantly reduced the frequency of seizures after positive pressure ventilation (Devinsky et al., 1994). It was further confirmed in a recent studies including 503 participants that OSAS frequently coexisted with epilepsy, and CPAP could reduce the frequency of seizures (Jo et al., 2022). It was also observed in 2008 that after left frontal lobectomy was performed in patients with epilepsy complicated with OSAS, the seizure almost disappeared due to resolution of obstructive sleep apnea (Foldvary-Schaefer et al., 2008). Meanwhile, epilepsy patients often suffer from various sleep disorders, and treatment for OSAS can significantly improve epilepsy seizures in patients (van Golde et al., 2011). However, there is insufficient evidence regarding the mechanism of epilepsy’s impact on OSAS at present, which needs to be further discovered.

## 4 Intermittent hypoxia: linkage between OSAS and epilepsy

Recurrent upper respiratory tract obstruction in sleep apnea syndrome can lead to hypoxia of arterial microcirculation and proliferation of capillary endothelial cells, resulting in autonomic nervous dysfunction and increased the risk of epilepsy (Valli et al., 1984). It was noted that patients with epilepsy with or without OSAS will have a state of hypoxia. Whether epileptic patients are complicated with OSAS or not, the causes of hypoxia will be different. Patients with simple epilepsy will have a state of hypoxia during seizures, but patients with both epilepsy and OSAS will not only have hypoxia during seizures, but also have intermittent repeated hypoxia due to sleep apnea (Xanthopoulos et al., 2015; Tereshchenko et al., 2019; Han et al., 2020). It was observed that mice developed shortness of breath during seizures, followed by shallow breathing and hyperventilation (Trosclair et al., 2020). Central and obstructive apnea can also occur during seizures (Nakase et al., 2016). These symptoms may lead to epilepsy-related hypoxemia asphyxia and even sudden death in severe cases, which suggests a potential connection between epilepsy and OSAS (Bateman et al., 2008). It was also demonstrated in a meta-analysis that 33.4% of patients with moderate to severe epilepsy had co-existing OSAS, and patients with epilepsy were more likely to develop OSAS than healthy controls (Lin et al., 2017). At the same time, there is also evidence indicating that OSAS can trigger epilepsy. In a clinical study involving 25 pediatric patients with OSAS, overnight EEG monitoring revealed epileptiform brain discharges coinciding with OSAS onset in 2 children, and seizures in 2 other children, indicating that there may be a two-way relationship between OSAS and epilepsy (Miano et al., 2010; Brunetti et al., 2017).

Hypoxia may also lead to seizures. Current international academic research on hypoxia and epilepsy susceptibility is insufficient. However, a study indicates that closing a patent foramen ovale improves migraine and epilepsy by reducing hypoxia induced by right-to-left shunts (Dong et al., 2023). Another epidemiological study revealed that hypoxia in middle-aged adults with sleep apnea significantly increased the risk of late-onset epilepsy (Grigg-Damberger and Foldvary-Schaefer, 2023). Currently, the primary research on hypoxia-induced epilepsy is focused in the field of children. Neonatal rat models of mild hypoxia causing epilepsy without significant brain damage have been established (Justice and Sanchez, 2018). Researchers have attempted to control hypoxic-induced neonatal epilepsy in rats through a variety of approaches, and it was demonstrated that seizure susceptibility is associated with brain damages in male mice treated with hypothermia after neonatal hypoxia ischemia (McNally et al., 2019). Another study demonstrated that administering fingolimod, a sphingosine 1-phosphate receptor (S1PR) modulator known for its anti-inflammatory effects in the central nervous system (CNS), after hypoxia-induced neonatal seizures, successfully reversed cognitive impairment and reduced seizure severity in both male and female adult rats (Mehling et al., 2011; Najafian et al., 2021). Meanwhile, some medications can reduce the susceptibility to epilepsy by inhibiting hypoxia. It was demonstrated that bumetanide can reduce the susceptibility to epilepsy by inhibiting abnormal hippocampal neurogenesis in newborn rats after hypoxia ischemia (Hu et al., 2017). Taurine deoxycholic acid (TUDCA) improves the pathological changes of epileptic seizures in rats by reducing inflammation and hypoxia in autonomic nervous tissue (Üner et al., 2023). Minocycline inhibits the activation of microglia induced by hypoxia and reduces the onset of epilepsy (Fukushi et al., 2023). Therefore, there might be a causal relationship between OSAS and epilepsy.

### 4.1 Different types of hypoxia and brain excitability

Different types of hypoxia have different effects on brain excitability. Studies have shown that chronic and persistent hypoxia triggers the activation of microglia, and enhances glutamatergic neurotransmission in the NTS at the same time (Lima-Silveira et al., 2019). It was demonstrated by Wais M *et al.* that repeated hypoxic episode could induce seizures with alterations in hippocampal network activities (Wais et al., 2009). Another study showed that chronic, persistent hypoxia enhances neuroplasticity (Kuloglu et al., 2021). However, chronic intermittent hypoxia in the brain led to low-grade neuroinflammation in the hippocampus of mice, involving early but temporary cytokine elevation and delayed but long-term microglial changes. These changes may lead to intermittent hypoxia-induced cognitive impairment and pathological brain aging (Sapin et al., 2015). Compared with chronic hypoxia, acute hypoxia can cause severe and irreversible damage to brain function and eventually lead to cortical neuron death (Zhang et al., 2023).

Hypoxia has different effects on brain excitability at different ages. It has been demonstrated that specific groups of neurons and regions in the developing brain (cortex, thalamus, and putamen) are particularly sensitive to hypoxia during the neonatal period, a phenomenon known as selective vulnerability (Johnston, 1998; Johnston and Hoon, 2000). Damages caused by hypoxia can last for months or even years, causing mitochondrial failure, acute inflammation, oxidative stress and increased seizure activity (Fleiss and Gressens, 2012). It was noted by Rakhade SN *et al.* that hypoxia-induced neonatal seizures could result in the development of epilepsy in later life (Rakhade et al., 2011). In adults, hypoxia is a cause of neurological damage, including triggers for Alzheimer’s, Parkinson’s, and other age-related neurodegenerative diseases, and can also be a neuroprotector, a potential therapeutic application for neurodegenerative diseases (Burtscher et al., 2021).

### 4.2 Intermittent hypoxia: primary outcomes of OSAS

**FIGURE 1:**
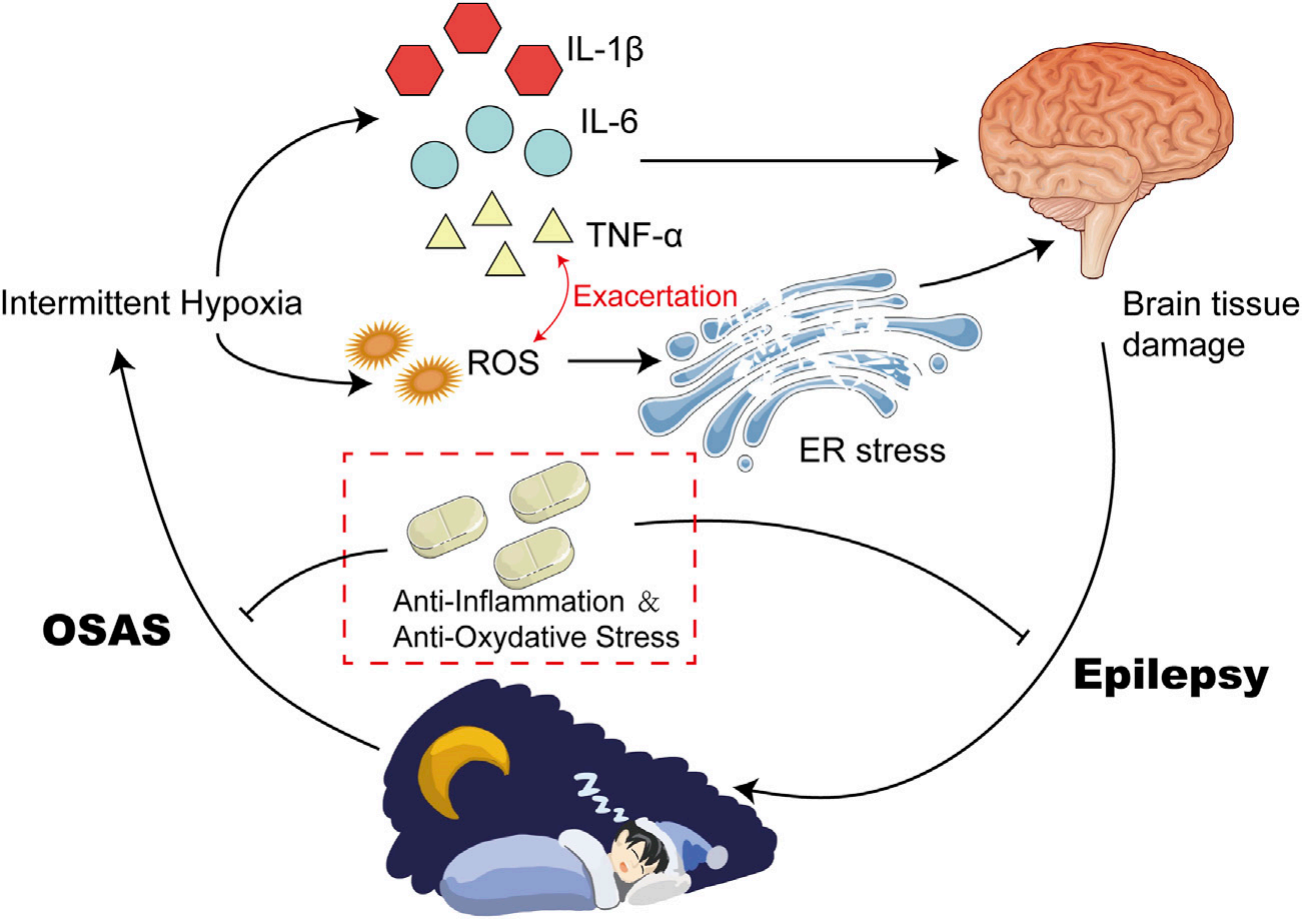
Intermittent Hypoxia Could Result in High Levels of Inflammatory and ROS. Intermittent Hypoxia could upregulate the expression of HIF-α via increasing its posttranslational stabilization. Elevated HIF-α induced the transcription of various inflammatory cytokines, such as IL-6 and TNF-α. In addition, hypoxia can also disrupt the balance of antioxidants between the defense system and the oxidative system, leading to upregulation of ROS. Elevated IL-6 and TNF-α exacerbate each other, and finally contribute to high levels of inflammation and oxidative stress.

As is mentioned above, OSAS is a severe sleep-disordered breathing disorder in which patients are intermittently hypoxic due to repeated apneas and hypoventilations during sleep (Benjafield et al., 2019). More and more studies have shown that OSAS patients often experience long-term intermittent hypoxia due to changes in blood gas balance caused by upper airway collapse and apnea/hypopnea events (Andaku et al., 2015). The pathogenesis of OSAS is multifactorial with multiple mechanisms, including oxidative stress, inflammation, endothelial dysfunction, and altered metabolism (Aherne and Hull, 1966; Salman et al., 2020). Intermittent hypoxia is a major pathophysiological feature of OSAS, which could trigger a variety of pathological states including oxidative stress, over inflammation, apoptosis, and increased neural activation (Patke et al., 2020) (Figure 1).

### 4.3 Intermittent hypoxia results in oxidative stress

Studies have shown that oxidative stress plays an important role in neurological injury caused by intermittent hypoxia (Zhang and Veasey, 2012; Lavie, 2015). Oxidative stress is a state of imbalance between pro-oxidant and anti-aging agents caused by various endogenous or exogenous stimuli or stressors, which is induced by overproduction of reactive oxygen species (ROS) and reactive nitrogen or a reduction of antioxidants (van der Pol et al., 2019). ROS is a double-edged sword. Mild levels of ROS can facilitate the activation of signaling pathways associated with repair and survival, while excessive levels can lead to adverse effects and inflammation (Tang et al., 2019). Hypoxia-induced elevated production of ROS can be attributed to several factors, including mitochondrial dysfunction, activation of NADPH oxidase (NOX) and xanthine oxidase, and uncoupling of nitric oxide synthase (NOS) (Lavie, 2003; Lavie, 2015). A large number of signaling pathways and transcription factors are regulated by ROS, the most notable of which are the Hypoxia-Inducible Factor 1α (HIF-1α) and nuclear factor-κB (NF-κB) pathways in OSAS (Reuter et al., 2010). NF-κB is a typical pro-inflammatory signaling molecule (Lawrence, 2009). NF-κB serves as an important regulator of various inflammatory processes by activating molecules that induce the production of pro-inflammatory cytokines such as tumor necrosis factor-α (TNF-α), IL-1β, and IL-6, along with chemokines and adhesion molecules (Shih et al., 2015).

Hypoxia in patients with OSAS can cause an imbalance of antioxidants between the defense system and the oxidative system, leading to oxidative stress, which in turn activates and accelerates peroxidative damage (Lavie, 2015). It was observed that circulating markers of protein nutrition and lipid peroxidation are elevated in OSAS patients, and the severity is related to the apnea-hypopnea index (Klein et al., 2010; Vatansever et al., 2011). Biomarker studies in OSAS patients have shown that ROS could reduce plasma nitrite and nitrate levels, increase lipid peroxidation, and reduce antioxidant capacity (Kanbayashi et al., 2009). Furthermore, oxidative stress in OSAS is associated with surrogate markers of cardiovascular disease, such as endothelial function, intima-media thickness, and hypertension (Eisele et al., 2015). In addition, OSAS patients exhibited alterations in metabolomic results which appear to be attributable to oxidative stress induced by OSAS (Xu et al., 2016) (Figure 1).

### 4.4 Intermittent hypoxia results in inflammation

Inflammation is the body biological response to injury, infection, or other stimuli, which is controlled by the body immune system and is designed to protect the body from harm (Gopal, 2020; Shen et al., 2020). The brain consumes a greater amount of oxygen and energy compared to the rest of the body, and is highly susceptible to hypoxia (Wang et al., 2010). Inflammation triggered by hypoxia can result in endothelial dysfunction and atherosclerosis in the brain, leading to a drop in cerebral blood flow and decreased neuronal metabolic function and oxygen consumption, which subsequently result in nerve cell apoptosis and necrosis (Zhang et al., 2016). Inflammation occurred in the peripheral regions of the body could reach the central nervous system through either blood-brain barrier penetration or vagal afferent stimulation (Dilger and Johnson, 2008). High levels of inflammation in the central nervous system further upregulate the activity of glial cells (microglia and astrocytes), inducing and exacerbating neuroinflammatory responses. At the same time, intermittent hypoxia can directly activate microglia and astrocytes, and promote the release of inflammatory cytokines in the central nervous system (Kiernan et al., 2016). Elevated levels of inflammation in the central nervous system can cause an increase in the activity of glial cells such as microglia and astrocytes, leading to the aggravation of neuroinflammatory responses (Neher et al., 2012; Dang et al., 2021).

Additionally, intermittent hypoxia can directly stimulate microglia and astrocytes, resulting in the release of inflammatory factors within the central nervous system (Hong et al., 2016). Inflammatory factors are molecules that mediate and regulate inflammatory responses during inflammation, including cytokines, chemokines, inflammatory mediators, *et al.* Inflammatory factors can promote or inhibit inflammatory responses by regulating the activity and proliferation of inflammatory cells (Shen et al., 2020). Excessive production of inflammatory factors under inflammatory conditions can lead to adverse effects, such as tissue damage and disease development (Jia et al., 2016). Intermittent hypoxia affects the occurrence and development of inflammation by regulating the production and differentiation of neutrophils, macrophages, and T cells (Hafner et al., 2017). Hypoxia could upregulate HIF1-α via increasing posttranslational stabilization (Meretoja et al., 2013). HIF1-α is a transcription factor which induces the transcription of multiple inflammatory factors via binding to hypoxia-response element (HRE) of target region, such as TNF-α and IL-6 (Wang et al., 2012; Supriya et al., 2016; Peek et al., 2017; Li et al., 2020). In addition, HIF1-α could also promote the expression of TNF-α and IL-6 via upregulation of NF-κB pathway (Bradshaw et al., 2020).

Increasing evidence supports OSAS as a low-grade chronic inflammatory disease. Many studies have confirmed that intermittent hypoxia and sleep deprivation are associated with inflammatory activation and progression in patients with OSAS (Biddlestone et al., 2015; Colgan et al., 2016; Nunes et al., 2018). There is a strong link between hypoxia and inflammation. It was observed that the levels of inflammatory factors in patients with OSAS were significantly higher than those in the control group (Kohler et al., 2010; Imani et al., 2020). Among these inflammatory mediators and pro-inflammatory cytokines, the most documented are IL-6 and TNF-α (Imani et al., 2020) (Figure 1).

#### 4.4.1 TNF-α

Tumor necrosis factor-α (TNF-α) is a widely studied inflammatory factor. TNF-α leads to activation of the NF-κB pathway and further activates nitric oxide synthase, cyclooxygenase and other receptors, participating in sleep regulation (Ramesh et al., 2012). Elevated levels of TNF- α were observed in OSAS patients, which were demonstrated to be associated with poor outcomes (Xin et al., 2022). In addition, it was noted by Olszewska E *et al.* that high levels of TNF- α were declined in OSAS patients after surgery (Olszewska et al., 2022). It was further reinforced in an animal experiment that chronic intermittent hypoxia induced by OSAS on mice result in high expression of TLR4-NFκB pathway and the increase of TNF- α level under hypoxia (Ramesh et al., 2012).

#### 4.4.2 IL-6

IL-6 is part of a group of cytokines known as the IL-6 family, which includes other members such as cardiotrophin-1, inhibin M, and leukemia inhibitory factor (Morris et al., 2018). Studies have shown that intermittent hypoxia induces macrophage polarization and increased IL-6 production (Li et al., 2017). Biopsies of adipose tissue and blood samples from obese patients with and without OSAS found that significantly increased tissue expression and circulating levels of IL-6, which was significantly attenuated after 6 months of continuous positive airway pressure treatment (Baessler et al., 2013). A systematic review examining inflammation and sleep disorders noted that two inflammatory factors, C-reactive protein (CRP), and IL-6, are strongly associated with sleep disorders, which upregulate the expression of NF-κB and increase the levels of intercellular adhesion molecules (Devaraj et al., 2006). At the same time, the combination of TNF-α and tumor necrosis factor receptor protein 1 (TNF-r1) can also increase the activity of NF-κB, resulting in increased expression of vascular cell adhesion molecule-1 and monocyte chemoattractant protein-1 in endothelial cells (Feng et al., 2018).

#### 4.4.3 IL-1β

IL-1β, one of the first interleukins discovered, produces a similar pro-inflammatory response through signaling of the interleukin-1 receptor type 1 (IL-1R1), which is involved in regulating the innate immune response of the host (Dinarello, 2011). The central role of IL-1β in mediating neuroinflammation has been recognized in most central nervous system-related diseases, activating microglia and astrocytes, and leading to downstream synthesis of other pro-inflammatory and chemoattractants within the CNS (Shaftel et al., 2008). IL-1β activates the IL-1R1-TLR4 signaling pathway by binding to IL-1R1, which can decrease the expression of GABA receptor and increase the expression of N-methyl-D-aspartate (NMDA) receptor in neurons and glial cells (Dheer et al., 2018; Cramer et al., 2019; Sanz and Garcia-Gimeno, 2020; Semple et al., 2020). One study shows that activation of IL-1R1-TLR4 signaling enhances the function of NMDA receptors in cultured hippocampal neurons by non-transcriptional mechanisms, thereby promoting NMDA-induced calcium influx and the release of excitatory neurotransmitters such as glutamate (Vezzani et al., 2011a). This suggests that the activation of this signaling pathway disrupts the homeostasis of various neurotransmitters and the excitability of neurons (Lai et al., 2006). Interestingly, IL-1β was found to enhance the releases of both glutamate and γ-aminobutyric acid (GABA) in the hippocampus through a mechanism engaging the toxic overload response of Ca^2+^ influx and Ca^2+^-induced Ca^2+^ release revealing that the IL-1β hyperactivation might cause an imbalance between excitatory and inhibitory neurotransmission (Zhu et al., 2006). Indeed, IL-1β was able to enhance the glutamatergic NMDA receptor through facilitating the Ca^2+^ influx, which was followed by increased neuronal cell death as a consequence of excitotoxicity (Viviani et al., 2003). These findings together suggest that IL-1β signaling via IL-1R contributes to the CNS hyperexcitability and excitotoxicity under neuropathophysiological conditions such as seizures likely through disrupting the balance between glutamatergic and GABAergic neurotransmission.

### 4.5 Oxidative stress and inflammation are mutually reinforcing

Oxidative stress and inflammation are two prominent potential mechanisms explaining various complications of OSAS. The former refers to a state where there is an unequal balance between pro-oxidative and anti-oxidative systems, resulting in the excessive production of ROS. The latter is response to various external and internal damages including oxidative stress. Inflammation is a response to oxidative stress, while oxidative stress and inflammation can mutually amplify each other (Wang et al., 2019). Oxidative stress sets off a harmful cycle, which triggers sympathetic activation and inflammation, thereby augmenting oxidative stress. ROS-activated PI3K/PKB signaling pathway mediates NF-kB activation, which is responsible for the release of inflammatory factors (Xu et al., 2004; Ravenna et al., 2014). In addition, NF-κB can also activate the transcriptional reaction of HIF-1α mRNA and serves as a prominent transcriptional activator of HIF-1α (Chou et al., 2016). Activation of HIF1-α could also stimulate expression of many proinflammatory molecules and exacerbate inflammation (Preston et al., 2019) (Figure 1).

## 5 Oxidative stress and inflammation could induce seizures

Epilepsy is a severe and persistent neurological disorder that is distinguished by the recurring, spontaneous onset of epileptic seizures. The primary cause of seizures is an imbalance between excitation and inhibition in the brain (Noam et al., 2013). This imbalance can result in the transition from normal brain function to an epileptic state. Epileptic activity can be promptly induced by the inhibition of synaptic and voltage-gated inhibitory conductances. Alternatively, it can be stimulated by the activation of synaptic and voltage-gated excitatory conductance (Matsumoto and Ajmonemarsan, 1964). A broad spectrum of theories has emerged from existing evidence to elucidate the pathogenic mechanisms of epilepsy. These theories encompass a range of factors such as modifications to ion channels, aberrant neurotransmitter release or uptake, metabolic dysfunction, inflammatory responses, neuronal loss, structural abnormalities, genetic factors, and more (Treiman, 2001; Kovac et al., 2017; Schmeiser et al., 2017; Terrone et al., 2017; Wei et al., 2017). Despite the numerous theories proposed, our understanding of the fundamental pathogenesis of epilepsy remains incomplete.

### 5.1 Inflammation induced by OSAS: a potent mechanism for epilepsy development

**FIGURE 2:**
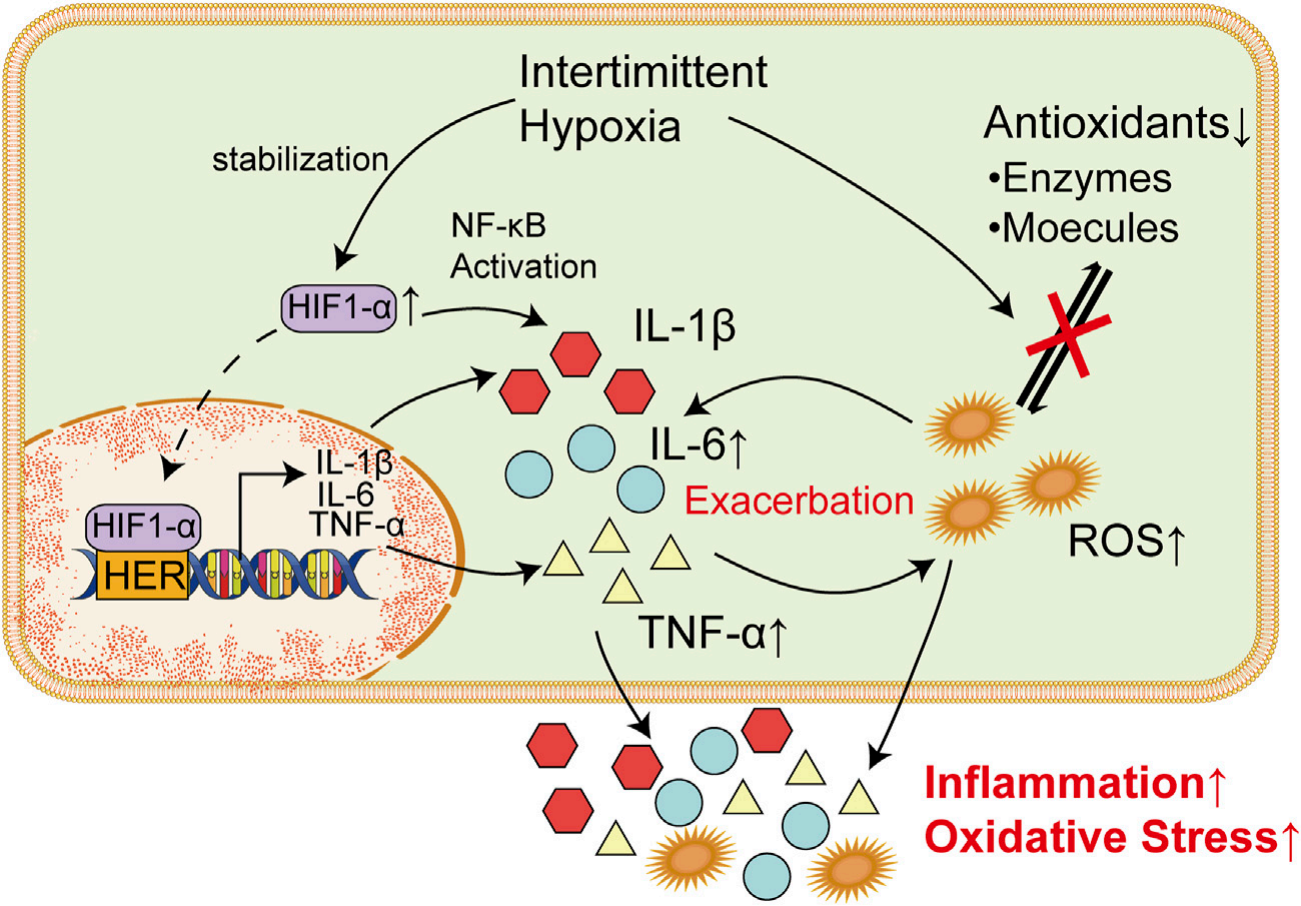
Inflammatory factors and ROS Are Responsible for Damages in Nervous Systems. Increased TNF-α could bind to corresponding receptor in microglia cells and trigger the release of glutamate via upregulating intracellular ATP/ADP and Ca^2+^. Elevated glutamate could thereby promote synaptic activity and lead to epilepsy. Meanwhile, IL-6 could increase the excitation of synapses when it binds to IL-6R through the JAK/STAT pathway. In endoplasmic reticulum, excessive ROS could lead to the production of a massive amount of unfolded or misfolded proteins, which result in the dissociation of GRP78 from ER transmembrane proteins (PERK, IRE1, and ATF6). Overactivation of these proteins induce unfolded protein response and subsequently lead to ER stress. ROS-mediated UPR could finally induce the apoptosis of neurons and thereby increase incidence of epilepsy.

As is mentioned above, chronic inflammatory conditions caused by chronic intermittent hypoxia is common in brain tissues among patients with OSAS. In the last decade, a growing amount of both clinical and experimental data has bolstered the idea that inflammatory mechanisms occurring in the brain may play a crucial and ubiquitous role in the development and progression of seizures and epilepsy (Vezzani and Granata, 2005; Vezzani and Baram, 2007; Riazi et al., 2010). In the case of febrile seizures, it has been noted that an increase in the concentrations of pro-inflammatory agents is frequently observed during epileptic events (Dinarello, 2004). Moreover, research has shown that certain drug-resistant forms of epilepsy may respond positively to treatment with anti-inflammatory agents like steroids, suggesting that they possess anticonvulsant properties (Wirrell et al., 2005; Wheless et al., 2007). Therefore, inflammatory processes within the brain, which was induced by intermittent hypoxia among patients with, maybe a fundamental and critical mechanism involved in the pathophysiology of seizures and epilepsy (Figure 2).

#### 5.1.1 Influence of TNF-α on epilepsy

Previous studies have shown that inflammatory factors represented by IL-6 and TNF-α play an important role in the mechanism of epileptic seizure (Dey et al., 2016). Animal evidence suggests that there may be an interaction between increased levels of TNF-α during seizures and, in turn, increased levels of TNF-α that lead to seizures, and this interaction seems to be a vicious cycle (Shandra et al., 2002). Transgenic mice that constitutively overexpress TNF-α showed multiple symptoms including seizures, ataxia, and paresis, along with inflammation-associated pathological alterations within the brain (Probert et al., 1995). Intriguingly, computational modeling of neuron-glia interactions also showed that TNF-α overexpression led to seizure-like activity patterns (Savin et al., 2009). Similarly, rats that received TNF-α prior to amygdala kindling showed prolonged seizure-like discharges and increased power of β and γ bands when compared to control animals (Shandra et al., 2002). TNF-α plays its role *in vivo* by interacting with TNF receptor type 1 (TNFR1) and TNF receptor type 2 (TNFR2) (Salazar-Montes et al., 2006; Hop et al., 2017; Sun, 2017). Interestingly, seizures lead to expression of TNF-α in the hippocampus and increase seizure sensitivity through activation of TNFR1, while activation of TNFR2 plays an antiepileptic role *in vivo* (Weinberg et al., 2013), Lack of TNFR2 or both led to increased epilepsy sensitivity in mice (Balosso et al., 2005). This suggests that the role of TNF-α in the course of seizures may be bidirectional.

Studies have shown that TNF-α can cause the activation of microglia cells and accelerate the death of peripheral neurons, which plays an important role in the occurrence and progression of epilepsy (Zhao et al., 2018; Henning et al., 2023). TNFR1 in microglia cells is activated after binding with TNF-α, which leads to increases in intracellular ATP/ADP and Ca^2+^ and subsequently trigger the release of glutamate. Increased levels of glutamate could promote synaptic activity and leads to epilepsy (Bedner and Steinhäuser, 2019). Another study also supports this claim. To prevent the harmful effects of TNF-a, some authors have used anti-TNF-α monoclonal antibodies (Etanercepp) and non-specific TNF-α inhibitors such as dihydrothalidomide, nilotinib, and cannabinoids. All of these treatments have been shown to be successful in improving seizures in animal models of epilepsy (Ravizza and Vezzani, 2018). Another study showed that TNF-α converting enzyme can reduce the incidence of seizures and play a neuroprotective role (Meli et al., 2004). It was also noted by Ljiljana Nikolic *et al.* that P2Y1 receptor inhibitors, which is known to block the activities of TNF-α, could restore abnormal neural activity in the hippocampus and thereby reduce epilepsy seizures (Nikolic et al., 2018). Targeting to cut off the purine metabolism of TNF-α and microglia may be a new strategy for the treatment of epilepsy.

#### 5.1.2 Influence of IL-6 on epilepsy

IL-6 is a convulsion-promoting and neurotoxic cytokine that regulates neuronal and synaptic function in the central nervous system (Gruol, 2015). High levels of IL-6 was observed in patients with epilepsy, suggesting that IL-6 is important for the development of epilepsy (Sallmann et al., 2000; Liimatainen et al., 2009). When it binds to IL-6R and gp130, it acts either through the JAK/STAT trans-signaling pathway (Leo et al., 2020). Meanwhile, IL-6 could disrupt the balance between the inhibition and excitation of synapses via excessive activation of synapses (Garcia-Oscos et al., 2012). Intranasal administration of IL-6 could increase the severity of epilepsy induced by pentylenenetrazole (Kalueff et al., 2004). It was also observed in IL-6-deficient mice that significantly fewer behavioral seizures was developed compared with wild mice when they are infected with neurotropic virus (Libbey et al., 2011). Therefore, IL-6 system may also act as a novel target for anticonvulsant drugs. Some researchers have suggested that the use of anti-IL-6 monoclonal antibodies or the use of specific STAT3 inhibitors such as WP1066 may help reduce seizures (Cerri et al., 2017). These results suggest that a certain baseline level of IL-6 appears to be necessary to maintain the seizure threshold, and that higher expression of IL-6 may exacerbate seizures and associated neuropathological changes.

#### 5.1.3 Influence of IL-1β on epilepsy

IL-1β serves as the archetypal inflammatory cytokine. Its binding to the IL-1β receptor (IL-1R) activates NF-κB and three mitogen-activated protein kinase (MAPK) signaling pathways, which play a crucial role in cytokine production, upregulation of inflammatory genes, and the generation of reactive oxygen species (Dey et al., 2016; Patel et al., 2017). IL-1R rises sharply in epilepsy (Allan et al., 2005; Ravizza and Vezzani, 2006; Maroso et al., 2010). IL-1β is commonly elevated in the serum and cerebrospinal fluid (CSF) of patients with epilepsy (Bauer et al., 2009; Shi et al., 2017). Similarly, in animal models of kainic acid (KA)-induced seizures, upregulation of IL-1β mRNA was observed in multiple brain regions including hippocampus, cerebral cortex, thalamus, hypothalamus, and striatum (Minami et al., 1991). One study showed that high doses of IL-1β induced seizures in mice expressing the IL-1β receptor (Dubé et al., 2005). Meanwhile, exogenous IL-1β can increase the proportion of epilepsy in rats after prolonged febrile convulsion (Fukuda et al., 2014). Intrahippocampal administration of IL-1β before KA injection in rodents significantly enhanced seizure severity and duration (Vezzani et al., 2002), suggesting a convulsive role of IL-1β in acute seizures. Therefore, IL-β system may also act as a novel target for anticonvulsant drugs. One study uses an antagonist of the IL-1β receptor IL-1R to prevent susceptibility to chronic seizures (Fukuda et al., 2014). Several studies have used anakinra (an antagonist of the IL-1R1 receptor) or anti-IL-1β monoclonal antibodies in animal models, both of which have been shown to reduce the occurrence of epilepsy (Vezzani, 2015; Dey et al., 2016). Interestingly, the combination of IL-1β and IL-6 antibodies is effective in preventing chronic epilepsy (Pineda et al., 2013).

#### 5.1.4 ROS exacerbates inflammation in brain tissues

Oxidative stress can induce inflammation, and inflammation can also promote the production of ROS (Lv et al., 2016). Brain tissue is vulnerable to oxidative stress (Freitas, 2009). It was demonstrated that oxidative stress-related seizures change the activity of antioxidant enzymes and the ability of receptor binding (Freitas et al., 2005). TNF-α is the key to coordinate oxidative stress and inflammation in the central nervous system (Gustafson et al., 2012). When the oxygen supply to the brain is insufficient, a large amount of ROS was produced, which then promotes the release of TNF-α (Wall et al., 2015). As an inflammatory factor in the brain, TNF-α can also rapidly increase the production of ROS and cause apoptosis mediated by oxidative stress in brain microvascular endothelial cells (Basuroy et al., 2011). In addition to TNF-α, oxidative stress can also upregulate the expression of IL-6 (Cho et al., 2005). IL-6 can aggravate the inflammatory response of premature infants and aggravate hypoxia-induced brain damage (Dammann and Leviton, 2000). Increased expression of IL-6 is involved in various neurological or mental diseases such as stroke, depression, epilepsy, Alzheimer’s disease, autism, *et al* (Wei et al., 2013; Ting et al., 2020; Rahman et al., 2021; Soltani Khaboushan et al., 2022; Zhu et al., 2022).

#### 5.1.5 Recent perspectives on neuroinflammatory factors and epilepsy

The latest view is that neuroinflammation is not a direct cause of epilepsy, but a modulator of epilepsy and a disease-inducing factor. Studies have shown that an increase in inflammatory molecules in the brain alone does not cause epilepsy (Iori et al., 2016; Villasana-Salazar and Vezzani, 2023), and epilepsy does not go into remission after treatment with anti-inflammatory drugs (Villasana-Salazar and Vezzani, 2023) There is a lot of evidence that inflammation contributes to seizures and seizures (Choi and Koh, 2008; Chiang and Serhan, 2017; Mukhtar, 2020). Signaling downstream of inflammation can lead to neuronal damage, which can affect the clinical manifestations of pathology. Patients with autoimmune diseases and encephalitis characterized by severe neuroinflammatory responses often exhibit a high incidence of seizures (Michael and Solomon, 2012; Sakamoto et al., 2022). The hyperexcitability that leads to seizures is the result of an imbalance in glutamatergic and GABAergic signaling. Many inflammatory mediators released by activated microglia and astrocytes lower the seizure threshold by regulating glutamatergic and GABAergic signaling. For instance, increase in TNF-α leads to upregulation of AMPA receptors and endocytosis of GABAergic receptors. IL-1β upregulates NMDA receptors, enhances glutamate release, and inhibits GABA release. Similarly, IL-6 also can promote glutamate release to enhance neuronal excitability. Therefore, the reduced seizure threshold and uninduced seizures are caused by a number of pathological changes, rather than directly induced by neuroinflammatory factors (Chen et al., 2023).

### 5.2 Inflammation and oxidative stress induced by OSAS: potential links to epilepsy via ER stress

The endoplasmic reticulum (ER) is a vital organelle in eukaryotic cells that performs several functions such as protein folding and quality control, cellular calcium ion homeostasis, and lipid biosynthesis (Anelli and Sitia, 2008; Song et al., 2017). However, various intracellular and extracellular factors such as hypoxia, oxidative injury, and mutant protein expression can disrupt ER function, leading to the accumulation of unfolded or misfolded proteins, which is termed as ER stress (Iurlaro and Muñoz-Pinedo, 2016; Hang et al., 2022). The degradation of misfolded proteins can start in the ER with the unfolded protein response (UPR) activation, which controls the stability of RNAs and the rate of protein synthesis, and activates the transcription of large amounts of genes (Yoshida, 2007; Cornejo et al., 2013; Coleman and Haller, 2019).

The UPR is a signal transduction pathway that involves stress sensors at the ER membrane and downstream transcription factors reprogramming gene expression towards either stress mitigation or the induction of proapoptotic programs (Chow et al., 2015). The three main ER transmembrane proteins, namely protein kinase R-like ER kinase (PERK), inositol-requiring enzyme 1 (IRE1), and activating transcription factor 6 (ATF6), initiate the UPR process (Hashimoto and Saido, 2018; Fu et al., 2020; He et al., 2020). PERK, IRE1, and ATF6 have both pro-apoptotic and protective functions, and there is an overlap amongst their activities (Fu et al., 2018; Balsa et al., 2019; Di Conza and Ho, 2020). Under normal conditions, the ER chaperone 78-kDa glucose-regulated protein (GRP78), inhibits the activation of these ER transmembrane proteins by binding to their lumenal domains (Bertolotti et al., 2000). However, under ER stress, the accumulation of misfolded and unfolded proteins could result in the dissociation of GRP78 from the lumenal domains, which subsequently lead to their activation (Lebeaupin et al., 2018). There is increasing evidence to suggest the crucial role of the ER stress and UPR in the pathogenesis of epilepsy (Yamamoto et al., 2006; Liu et al., 2019; Nowakowska et al., 2020). Moreover, interventions aimed at mitigating ER stress have demonstrated both antiepileptic and neuroprotective effects in epilepsy (Zhang et al., 2019; Fu et al., 2020). Thus, ER stress is considered as one of the predominant mechanisms in the pathogenesis of epilepsy (Figure 2).

#### 5.2.1 Role of ROS in exacerbating ER stress

As is mentioned above, inflammation and high levels of ROS is common among patients with OSAS due to intermittent hypoxia. Inflammation and ROS are also involved in the induction of ER stress, which contribute to epilepsy (Wang et al., 2014; Li et al., 2019).

Oxidative stress has the potential to disrupt endoplasmic reticulum (ER) function and trigger ER stress (Wang et al., 2017). In ER, ROS stimulate the activation of PERK, ATF-6α, and IRE1α, which increased abnormal protein folding and subsequently triggers endoplasmic reticulum (ER) stress (Zhang and Kaufman, 2008; Zhang et al., 2019). ROS-mediated UPR could lead to apoptosis through the activation of PERK (Hong et al., 2015). Meanwhile, upregulation of ATF-6α and IRE1α could also induce neuronal apoptosis (Li et al., 2013; Zhu et al., 2020). Neuronal apoptosis is a common outcome induced by seizures, which could lead to epileptogenesis and cognitive impairment (Bengzon et al., 2002; Henshall and Simon, 2005) (Figure 2).

#### 5.2.2 Role of inflammation in exacerbating ER stress

Numerous studies have shown that inflammation is connected with ER stress, which is triggered by the activation of the NF-κB signaling pathway and the release of various proinflammatory cytokines (Dong et al., 2016). It was also noted *in vivo.* That exposure to IL-6 could reduce calcium storage of ER, activate UPR and thereby increased cell death (O'Neill et al., 2013). Notably, ER stress and inflammation are two-way interactions (Liu et al., 2012). Activated IRE-1 caused by UPR could recruit TRAF2, which result in activation of downstream signaling of kinases and upregulate the expression of various inflammatory cytokines, such as TNF-α and IL-6 (Amen et al., 2019). These inflammatory cytokines could in turn exacerbate ER stress and result in neuronal apoptosis (Figure 2).

## 6 Discussion

**FIGURE 3:**
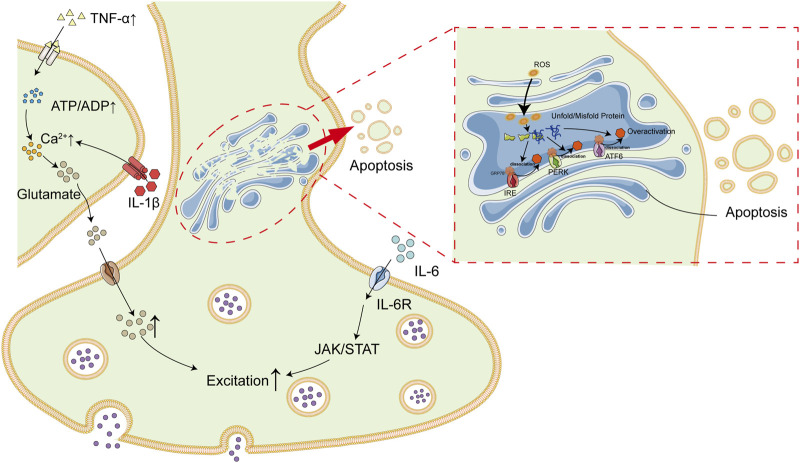
Association Between OSAS and Epilepsy. Patients with OSAS are in a state of long-term intermittent hypoxia due to upper airway collapse. Intermittent hypoxia result in high levels of inflammatory cytokines (e.g., IL-6 and TNF-α) and ROS, which subsequently lead to inflammatory and ER stress in brain tissues. In addition, IL-6 and TNF-α could also exacerbate each other. All of these deregulations finally contribute to high incidence of epilepsy among patients with OSAS.

Emerging studies suggest that patients with OSAS are predisposed to injuries in nervous system. It is proposed in this study that intermittent hypoxia is the linkage between OSAS and epilepsy. Long-term intermittent hypoxia is common among patients with OSAS during sleep due to upper-airway collapsibility, which lead to high levels of oxidative stress and inflammation. ROS and inflammatory factors, primarily TNF-α and IL-6, result in nervous system insults via induction of inflammation and ER stress in brain tissues, which subsequently increase the risks of epilepsy (Figure 3).

Various drugs targeting inflammation and oxidative exhibit therapeutic effects on OSAS and epilepsy. Melatonin, which have broad effects in anti-inflammatory and anti-oxidative stress, was confirmed to have antiepileptic effects as an antioxidant and neuroprotective drug (Park et al., 2015; Khan et al., 2020). In addition, anti-inflammatory therapy is also an effective treatment to reduce seizures and also improve the prognosis (Gershen et al., 2015; Fan et al., 2019). ACTH, steroids and intravenous immunoglobulin (IVIg) have been used in the treatment of seizures (Vezzani et al., 2011b). It was also observed that various anti-inflammatory drugs have therapeutic effects on drug-resistant epilepsy through specific inflammatory pathways in epilepsy (Terrone et al., 2017). In addition, non-steroidal anti-inflammatory drugs have also been used in 2013 as a feasible choice of antiepileptic drugs (Yu et al., 2013).

Recently, there has been an increasing interest in the nervous system impairments among OSAS patients. OSAS could decrease the seizure threshold in epilepsy patients and thereby increase the incidence of epilepsy seizures (van Golde et al., 2011). In this study, we proposed that intermittent hypoxia caused by OSAS is a critical factor in the pathogenesis of epilepsy via upregulation of oxidative stress and inflammation levels. However, there have been relatively few studies focusing on the role of intermittent hypoxia in the relationship of OSAS and epilepsy. Therefore, greater focus on the status of hypoxia maybe warranted in those with OSAS to prevent and reduce damage to the nervous system. Researchers should first pay more attention to the sleep quality of epilepsy patients to confirm the existence of hypoxia. Next, it is necessary to investigate whether resolving intermittent hypoxia in epilepsy patients during sleep could result in a reduction of epilepsy symptoms. In addition, more studies are also required to further understand mechanisms linking OSAS and epilepsy, such as oxidative stress, inflammation, and ER stress, which might provide novel therapeutic targets on epilepsy.
